# Mushroom Poisoning: A Rare Etiology of Acute Liver Failure

**DOI:** 10.7759/cureus.51144

**Published:** 2023-12-27

**Authors:** Hassan Mohammed Ismael Mohammed, Farooq Ahmad

**Affiliations:** 1 Respiratory Medicine, University Hospital of North Durham, Durham, GBR; 2 Internal Medicine, Grantham and District Hospital, Grantham, GBR

**Keywords:** n-acetyl cysteine, amatoxin poisoning, amanitn phalloides, orthotropic liver transplantation, acute liver failure (alf), mushroom poisoning

## Abstract

Acute liver failure is defined as a rapid deterioration in liver function, manifested by symptoms and signs of hepatic encephalopathy and disturbed synthetic function in a patient without Pre-existing cirrhosis and with an illness of less than 26 weeks duration. Mushroom poisoning as a cause of acute liver injury is rare but associated with deadly outcomes if not early recognized and treated. The mortality is very high in the case of amatoxin-containing mushrooms ingestion and liver transplantation is the only lifesaving option. Therefore, early recognition of a suspected patient who came with features of mushroom-related food poisoning, timely referral to a liver transplantation center, and adequate supportive management remain the main approaches of management in a patient with acute liver injury. We present a patient with gastroenteritis who ingested wild mushroom 14 hours prior to hospital admission with subsequent severe acute liver failure due to mushroom poisoning, successfully treated with urgent liver transplantation. This case study highlighted that careful evaluation of the symptoms and signs of acute liver failure in a patient with a history of mushroom ingestion can result in early referral to a liver transplant center, especially if the patient is systemically unwell.

## Introduction

Mushroom poisoning [1] is rare but is one of the deadly causes of acute liver failure (ALF). Ingestion of the poisonous mushroom, called Amanita phalloides, is one of the most common causes of mushroom poisoning worldwide, being involved in various countries all over the world. The mechanism of mushroom poisoning related to liver damage by Amatoxin poisoning [2] is due to a toxin known as amanitins. The toxic effect can lead to impairment in protein synthesis by the inhibition of RNA polymerase II, resulting in subsequent hepatocellular necrosis, acute liver injury, and death if left untreated with urgent liver transplantation. Initially, most of the patients are asymptomatic within the first six to 24 hours of mushroom poisoning, called lag phase, usually followed by symptoms that resemble viral gastroenteritis, mainly diarrhea and vomiting, with subsequent rapid development of acute severe liver and kidney injuries requiring fluid resuscitation.

According to the data around the globe, it is difficult to identify the exact mushroom species on initial presentation in the majority of mushroom poisoning, and therefore, the working diagnosis is based on detailed history taking, especially if there is any history of ingestion of wild mushroom prior to the onset of the symptoms and on the clinical manifestation at the time of hospitalization. This case report illustrates the importance of early involvement of different specialties in the case of acute liver failure as well as early recognition of the patient who might need transfer to a tertiary center for liver transplantation as the only life-saving option is very essential.

## Case presentation

We presented a case of a 51-year-old woman, who attended to accident and emergency department (A/E) of our hospital with diarrhea, vomiting, and abdominal pain which had been present for six hours prior to hospital admission. Apart from mild dehydration and right upper quadrant abdominal tenderness, her physical examination did not detect other abnormalities. She had been diagnosed by the A/E team with viral gastroenteritis, based on initial blood test results which showed mildly deranged liver and renal functions for which she was referred to the medical team for intravenous fluid and other symptomatic management (Table 1).

**Table 1:** Patient's initial blood test result on admission to hospital.

Blood test	Normal ranges	Test result
ALT	7 to 55 units per liter (U/L)	67
AST	8 to 48 U/L	72
ALP	40 to 129 U/L	130
Albumin	3.5 to 5.0 grams per deciliter (g/dL)	4
Total protein	6.3 to 7.9 g/dL	6.5
Bilirubin	2 to 17 micromoles/L	22
GGT	8 to 61 U/L	51
PT	9.4 to 12.5 seconds	13
INR	1	1.3
Creatinine	Women 45–84 μmol/ L	53
eGFR	>90mL/min/1.73m^3^	>90
Urea	2.5–7.8 mmol/L	4.2
Sodium	135–146 mmol/L	138
Potassium	3.5–5.3 mmol/L	4.2
Hemoglobin	11.5–15.5 g/dL	13.7
Platelet count	150,000–400,000/mL	234,000
White blood cell	5,000–10,000/mL	11,500
CRP	C-reactive protein	37

Six hours later while awaiting a bed in the acute medical unit; she suddenly deteriorated and became confused and hypotensive. A medical emergency call was activated, and a systemic urgent clinical review confirmed a confused, restless, and clinically dehydrated patient. Repeat Blood gas showed profound acidosis with raised lactate of -7.9 mmol/L and hypoglycemia with blood glucose of 2.3 mmol/L. These raised lactate and profound hypoglycemia in otherwise fit and well patients prompted the treating team to repeat the blood test including, FBC, U/E, LFT, CRP, coagulation profile, blood/urine c/s for septic screening as well as cortisol level and CT head. The result of the blood test showed evidence of severe liver and kidney injuries, but her CT head report was normal (Table 2).

**Table 2:** Repeated blood test following deterioration of the patient's condition.

Blood test	Normal ranges	Test result
ALT	7 to 55 units per liter (U/L)	880
AST	8 to 48 U/L	1,250
ALP	40 to 129 U/L	341
Albumin	3.5 to 5.0 grams per deciliter (g/dL)	3.5
Total protein	6.3 to 7.9 g/dL	5.1
Bilirubin	2 to 17 micromoles/L	334
GGT	8 to 61 U/L	320
PT	9.4 to 12.5 seconds	23
INR	1	5.2
Creatinine	Women 45–84 μmol/ L	340
eGFR	>90mL/min/1.73m^3^	29
Urea	2.5–7.8 mmol/L	23.9
Sodium	135–146 mmol/L	145
Potassium	3.5–5.3 mmol/L	5.6
Hemoglobin	11.5–15.5 g/dL	14.1
Platelet Count	150,000–400,000/mL	180,000
White blood cell	5,000-10,000/mL	22,000
CRP	C-reactive protein	288

The patient was resuscitated with IV fluid and IV glucose, and she was put in a high-dependency area. Non-invasive liver screens including viral hepatitis along with paracetamol, acytel-salylate, and ammonia levels were also requested. Diagnosis of ALF was made at this stage, and the patient was started on N-acetyl-cysteine (150 mg/kg in 500 mL 5% dextrose over four hours, followed by 50 mg/kg over four hours then 50 mg/kg) [3] and intravenous Piperacillin with tazobactam 4.5g intravenous every eight hours as broad-spectrum antibiotics. Collateral history was taken from her partner, to figure out the possible cause of ALF and to exclude any contraindication for a liver transplant. The patient had no significant past medical or surgical history, no history of alcohol excess or any risk factors for viral hepatitis, and was not taking any medications, specifically paracetamol or acytel-salylate. However, he mentioned that they had eaten wild mushrooms around the area 14 hours prior to the onset of her symptoms. At this stage ALF secondary to mushroom poisoning as a cause of ALF was suspected. The case was discussed with the local liver transplant team and the patient was shifted to intensive care while awaiting the transfer process. The diagnosis was confirmed by measuring urinary amatoxin levels through a laboratory test. The next morning, the patient was transferred to the transplant unit, and luckily, she was transplanted as she was fulfilling the King’s College Hospital criteria for acute liver transplant. The transplantation was successful, and her liver function test had normalized only after eight weeks following the operation.

## Discussion

According to different global studies, there are more than 100,000 species of mushrooms worldwide but only 100 species are presumed to be poisonous [4]. The actual incidence of mushroom poisoning is not clearly estimated due to the fact that relatively high numbers of cases have not been reported; however, amatoxin poisoning is a worldwide problem. In Western Europe, approximately 100 fatal cases are reported every year [5]. Reported cases are less common in the United States [6], Africa, Asia, Australia, as well as Central and South America. Overall, studies have shown that in the case of poisonous mushroom ingestion, the mortality rate of the affected person is expected to be around 20% [6]. However, a systematic review and meta-analysis study showed only 2.87% (7) of the mortality rate in liver transplantation group [7]. The toxicity of amanitin remains effective whether eaten cooked or uncooked, the lethal dose may be as small as 7 mg in adult patients weighing 70 kg and this amount can be absorbed even by eating a single piece of mushroom [8]. The mechanism of action of amatoxin intoxication is caused by disruption of transcription of messenger RNA polymerase11, resulting in inability of the hepatic cell to synthesize key protein-coding genes, leading to the disintegration of nucleoli and pathologically centrilobular liver cell death and subsequent necrosis. This leads to the insidious onset of liver failure over 24 to 48 hours depending on the type of mushroom ingested. The clinical manifestation ranges from mild non-specific symptoms such as gastroenteritis with progressive major cytotoxic effects and multi-organ failure and eventually death if left untreated.

According to the literature review, the first stage of gastrointestinal symptoms in relation to amatoxin type of mushroom poisoning usually starts between six to 24 hours after ingestion of the mushroom. The typical symptoms consist of diarrhea, nausea, and vomiting which can progress to the right upper quadrant abdominal pain along with other systemic neuropsychiatric and dermatological manifestations.

It is important to consider this period as an alarming sign of mushroom poisoning in patients with a history of recent ingestion and supportive treatment should be commenced before the development of life-threatening events like liver and renal failure [9]. This period must be suspected in all patients with evidence of acute jaundice following an acute gastrointestinal episode, especially if there is a history of mushroom ingestion.

The diagnosis of mushroom poisoning is clinical in most cases, but the confirmatory test can be done by measuring urinary amatoxin levels and identification of the mushroom through laboratory tests, though is not available in all health facilities. For the cases presented with severe liver injuries and carrying poor prognosis, emergency liver translation is the only lifesaving treatment which can be either orthotopic type of liver transplantation (OLT) or auxiliary partial orthotopic liver transplantation (APOLT) [10]. The effects of other treatment options with combinations of various drugs [11] such as N-acetyl cysteine and penicillin-G along with other supportive therapies like artificial liver support systems (ALSS) remain limited [12]. The early identification of patients with amatoxin poisoning and improvements in the efficacy of specific therapies will benefit most of the patients with the diagnosis of ALF associated with mushroom poisoning. It is worth mentioning, that care and consideration should be given to other common causes of sudden devolvement of acute injuries to the liver in otherwise fit and well patients such as infection, acetaminophen overdose, and autoimmune disorders [13].

## Conclusions

This patient was referred to us for gastroenteritis, however the care evaluation of the symptoms and signs of ALF in the context of mushroom ingestion as well as early involvement of different specialties along with early recognition of the patient who might need urgent referral to the transplant unit, especially if the patient is systemically unwell result in successful urgent liver transplantation as the only lifesaving treatment. Although the mortality rate in patients with mushroom poisoning is significant, there has been improvement in the overall survival outcome through the early intensive care management approach along with emergency liver translation advancements. Although ALF secondary to mushroom poisoning is quite rare, clinicians must keep an open mind about the rare causes of ALF.
